# Species Association of Hepatitis B Virus (HBV) in Non-Human Apes; Evidence for Recombination between Gorilla and Chimpanzee Variants

**DOI:** 10.1371/journal.pone.0033430

**Published:** 2012-03-14

**Authors:** Sinéad Lyons, Colin Sharp, Matthew LeBreton, Cyrille F. Djoko, John A. Kiyang, Felix Lankester, Tafon G. Bibila, Ubald Tamoufé, Joseph Fair, Nathan D. Wolfe, Peter Simmonds

**Affiliations:** 1 Centre for Immunology, Infection and Evolution, University of Edinburgh, Edinburgh, United Kingdom; 2 Roslin Institute, University of Edinburgh, Midlothian, United Kingdom; 3 Limbe Wildlife Centre, Limbe, Cameroon; 4 Global Viral Forecasting, San Francisco, California, United States of America; 5 Global Viral Forecasting, Stanford University, Program in Human Biology, Stanford, California, United States of America; 6 Ape Action Africa, Yaounde, Cameroon; Blood Systems Research Institute, United States of America

## Abstract

Hepatitis B virus (HBV) infections are widely distributed in humans, infecting approximately one third of the world's population. HBV variants have also been detected and genetically characterised from Old World apes; *Gorilla gorilla* (gorilla), *Pan troglodytes* (chimpanzee), *Pongo pygmaeus* (orang-utan), *Nomascus nastusus* and *Hylobates pileatus* (gibbons) and from the New World monkey, *Lagothrix lagotricha* (woolly monkey). To investigate species-specificity and potential for cross species transmission of HBV between sympatric species of apes (such as gorillas and chimpanzees in Central Africa) or between humans and chimpanzees or gorillas, variants of HBV infecting captive wild-born non-human primates were genetically characterised. 9 of 62 chimpanzees (11.3%) and two from 11 gorillas (18%) were HBV-infected (15% combined frequency), while other Old world monkey species were negative. Complete genome sequences were obtained from six of the infected chimpanzee and both gorillas; those from *P. t .ellioti* grouped with previously characterised variants from this subspecies. However, variants recovered from *P. t. troglodytes* HBV variants also grouped within this clade, indicative of transmission between sub-species, forming a paraphyletic clade. The two gorilla viruses were phylogenetically distinct from chimpanzee and human variants although one showed evidence for a recombination event with a *P.t.e.*-derived HBV variant in the partial X and core gene region. Both of these observations provide evidence for circulation of HBV between different species and sub-species of non-human primates, a conclusion that differs from the hypothesis if of strict host specificity of HBV genotypes.

## Introduction

Hepatitis B virus (HBV) is a member of the *Hepadnaviridae* family of viruses, containing a partially double-stranded DNA genome of approximately 3182–3221 nucleotides [1]. Human hepatitis B virus is globally distributed, infecting approximately one third of the world's human population. A substantial proportion of liver disease is attributable to HBV, killing over one million people each year [2]. In South and East Asia, Sub-Saharan Africa and South and Central America populations show a particularly high frequency of HBV infection which can be maintained by vertical mother to child transmission or horizontal transmission during childhood [3].

HBV variants infecting humans show genetic and antigenic heterogeneity and are currently classified into 7 or 8 genotypes (A–H) with a nucleotide sequence divergence ranging from 9% to 13%. Two putative genotypes I and J have also been reported. Genotype I was tentatively suggested for strains recovered in Laos [4]. A ninth genotype J was recovered from an 88-year-old Japanese patient with hepatocellular carcinoma, with mean sequence divergence between HBV/J and gibbon and orangutan genotypes of 10.9% and 10.7% respectively [5].Both active and resolved HBV infections are also found at high frequencies in chimpanzees [6], [7] and South Asian apes [7]; whose 10–12 host taxa-associated variants are distinct from the human variants of the virus. In addition to the current HBV genotypes, recombination between human genotypes, for example between genotypes A and D [1], [8], [9], [10] and B and C [8], [9], [11], [12] can generate novel variants, contributing to the genetic diversity of the virus.

Within the past 10 years, both active and resolved HBV infections have also been found in chimpanzees [2], gorillas [13], gibbons [13], [14] and orang-utans [7] at infection frequencies comparable to human rates in endemic regions [15], [16] in addition to a single isolate from a woolly monkey [17]. Furthermore tentative evidence for the occurrence of recombination has been obtained between the human genotype C and the chimpanzee variant AF498266 [18] and gibbon variants [15]. Their occurrence demonstrates that despite their genetic divergence, human and non-human associated variants of HBV can share hosts in nature. A recently published study characterising HBV variants infecting ape populations in Cameroon [21] demonstrated the existence of a gorilla-specific HBV strain and evidence of recombination between HBV strains circulating in chimpanzees. This and previous studies of HBV nucleotide sequence similarity [14] indicate non-human primates (NHP) have distinct species-specific variants of HBV distinguishable both from each other and from human HBV despite occupying overlapping geographical areas.

Cameroon is within a region of endemic human HBV infection with a hepatitis B surface antigen (HBsAg) prevalence in humans of 8% or greater [20]. Additionally, four different great ape taxa also occur in Cameroon, providing the conditions for potential inter-species transmission. Although no human-derived genotypes of HBV were detected in non-human primates in the current study, evidence for transmission of HBV between chimpanzee subspecies and between chimpanzees and gorillas was obtained.

## Results

A total of 164 non-human primate plasma samples from 11 gorillas, 62 chimpanzees and 91 Old World Monkeys (OWM) were screened for the presence of HBV DNA. PCR screening showed 9/73 (12%) apes were positive corresponding to 2/11 gorillas (18%) and 7/62 chimpanzees (11.3%) and 2/91 (2.2%) OWM. Complete HBV genomes were obtained from the isolates of 2 *Gorilla gorilla* and 6 chimpanzees (4 *P.t.ellioti*, 2 *P.t.troglodytes*), while both the Old World Monkey isolates (1 Grey cheeked mangeby and 1 Mandrill) and 1 chimpanzee were positive only with the screening primers originally used.

**Figure 1:**
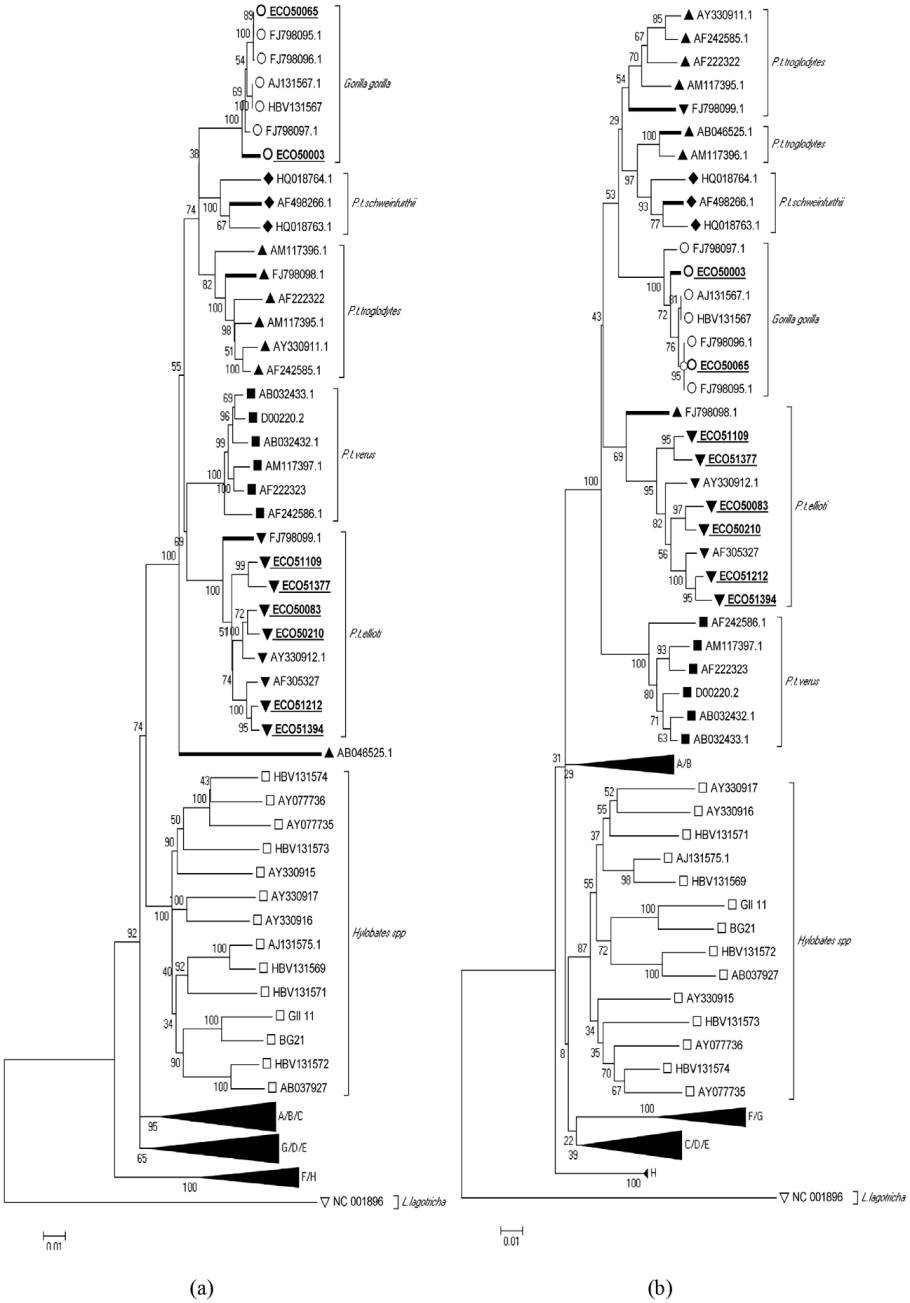
Phylogenetic analysis based on the HBV genome and identified recombinant region 1560–2120 bp. Phylograms displaying phylogenetic trees based on (a) complete HBV genome; (c) HBV recombinant region 1560–2120 bp and equivalent fragments immediately preceding; (b) 999–1559 bp and succeeding; (d) 2121–2681 bp this region; with HBV reference sequences from human genotypes (A–H). Relative species and sub-species HBV variants are identified as follows *Pan troglodytes troglodytes*▴, *Pan troglodytes ellioti*▾, *Pan troglodytes verus*▪, *Pan troglodytes schweinfurthii*♦, *Gorilla gorilla*○ and *Hylobates spp*. □, and the host specific cluster is identified by ]. The trees were rooted with the woolly monkey HBV sequence, NC_001896▿. Sequences from this study are in bold and underlined and while recombinant HBV variants have bold branches.

Phylogenetic analysis of the HBV strains using 415 bp S gene fragments confirmed the grouping of the novel chimpanzee HBV strains with previously published HBV chimpanzee sequences and the grouping of the two novel gorilla HBV sequences with previously published HBV strains AJ131657 and FJ98095-97 [19], [21]. (The novel HBV sequence ECO50065LIP3J and FJ98095 were retrospectively identified as originating from the same gorilla in Limbe Wildlife Centre) (Data not shown). Mitochondrial sequencing confirmed that the ECO50083LIP5, ECO50210LIP4, ECO51394CWP1.4, ECO51377CWP2 and ECO51109CWP4, ECO51212CWP6 HBV variants originated from chimpanzee subspecies *P. t. ellioti* and *P. t. troglodytes* respectively, while ECO50003LIP3 and ECO50065LIP3 were identified as gorilla-derived (Table 1). Complete genome sequencing of the eight study isolates produced sequences of 3182-bp in length, comparable to reference chimpanzee and gorilla strains (Fig 1). Phylogenetic analysis based on the complete genome (Fig. 1a), demonstrated monophyletic groupings for each human genotype (A–H), a clade containing gibbon and orangutans variants and a third containing chimpanzee and gorilla HBV sequences, each supported by high bootstrap values. Sequences of all novel chimpanzee HBV variants, irrespective of their sub species specific host, clustered with HBV sequences previously obtained from *P. t. ellioti* [21]. As the study samples were obtained from captive settings where *P. t. troglodytes* and *P. t. ellioti* are frequently co-housed, it remains unclear whether these findings reflect the HBV genotype distribution among wild chimpanzees in Cameroon or whether infection of *troglodytes* by *ellioti*-derived strains occurred in captivity.

**Table 1:** Specimen isolation data.

Specimen Number	Mito	HBV Variant	Location	Date arrival	Approx age on arrival	Date sample collection	Previous holder	Likely Wild Origin
Gorilla ECO50003LIP3	*G.g.*	*Gorilla gorilla*	Limbe Wildlife Centre	13-Sep-03	9 years	19-Aug-04	Nkoma, Mvangan, South Region	South Region
Gorilla ECO50065LIP3	*G.g*	*Gorilla gorilla*	Limbe Wildlife Centre	17-Dec-96	9 months	22-Jun-05	Bertoua, East Region	East Region
Chimpanzee ECO50083LIP5	*P.t.e.*	*Pan troglodytes ellioti*	Limbe Wildlife Centre	04-Oct-05	1.5 years	12-Oct-05	Garoua Zoo (North Region)	Adamaoua or East Region
Chimpanzee ECO50210LIP4	*P.t.e.*	*Pan troglodytes ellioti*	Limbe Wildlife Centre	31-Dec-04	8 months	05-Dec-06	Bachou, Manyu, South West Region	Banyang Mbo Widlife Sanctuary
Chimpanzee ECO51109CWP4	*P.t.t.*	*Pan troglodytes ellioti*	Mfou NP sanctuary	25-May-00	1.25 years	08-Aug-06	Mfou, Centre Region	Akom II, South Region
Chimpanzee ECO51212CWP6	*P.t.t.*	*Pan troglodytes ellioti*	Mfou NP sanctuary	08-Aug-06	1 years	10-Mar-08	Unknown	East Region
Chimpanzee ECO51394CWP1.4	*P.t.e.*	*Pan troglodytes ellioti*	Mfou NP sanctuary	13-Sep-05	1.5	26-Aug-09	Unknown	Centre Region
Chimpanzee ECO51377CWP2	*P.t.e.*	*Pan troglodytes ellioti*	Mfou NP sanctuary	01-Oct-97	4.6 yrs	26-Jul-09	Unknown	Unknown

**Figure 2:**
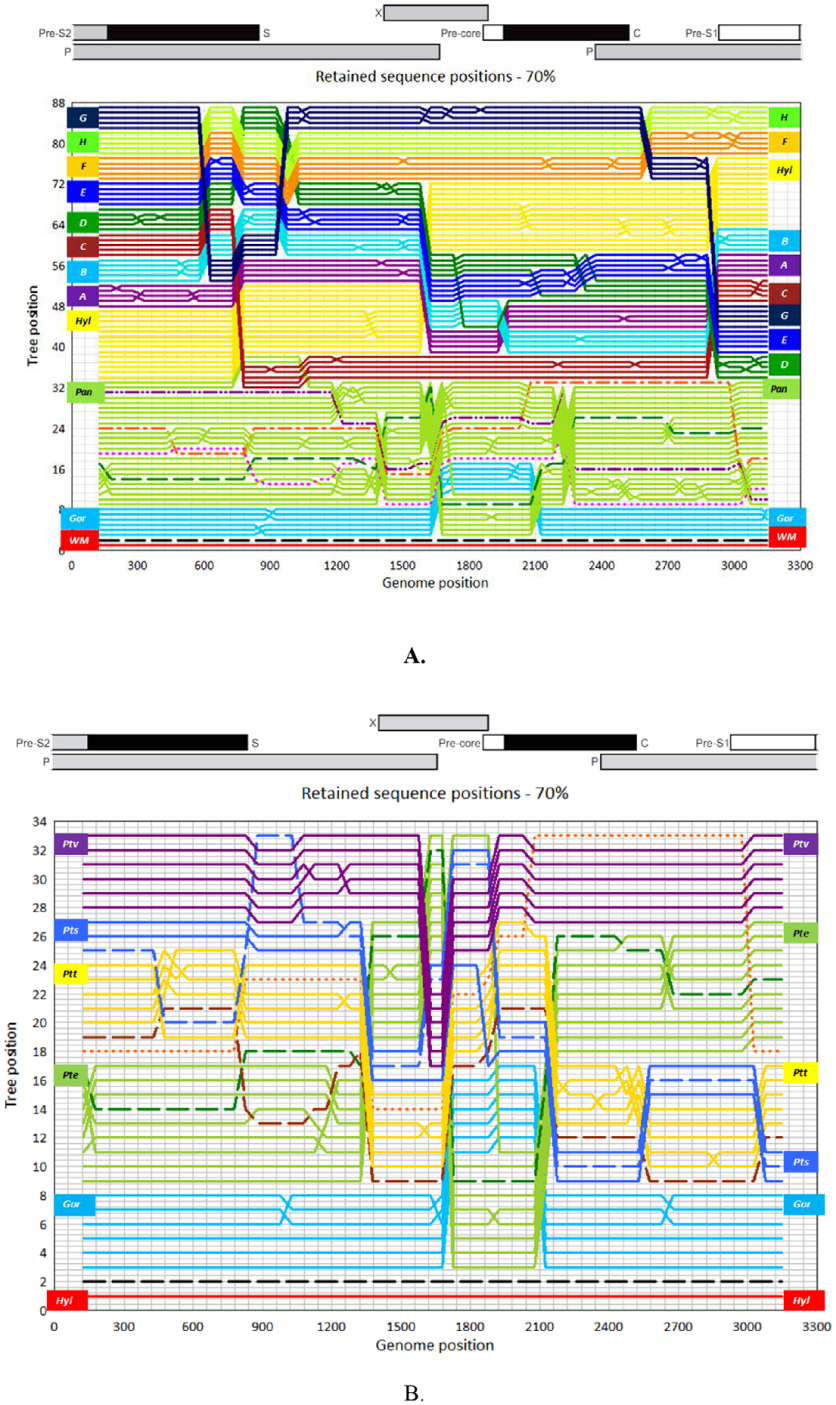
Tree Order Scan of HBV sequences. **Figure 2(a)**. TreeOrder Scan of HBV sequences, indicating positions of individual sequences (y axis) in Phylogenetic trees generated from sequential 250-base sequence fragments, incrementing by 50 bases. Changes in sequence order as a result of changes in phylogeny at the 70% bootstrap level are shown. Sequences are colour coded by genotype and host species, as indicated by the labels in left and right margin: genotype A, purple; B, light blue; C, wine; D, emerald; E, royal blue; F, orange; G, pale green; H, navy; Gorilla, blue (Gor); Chimpanzee, green (Pan); and Woolly monkey (WM-out-group on line 1), red. For comparison the Tree Order Scan has been aligned with scale genome of HBV (top panel). Recombinant sequences are highlighted as by dashed lines; black gorilla/*P.t.e* ECO50003LIP3, green FJ798099 *P.t.e/P.t.t*, pink FJ798098 *P.t.e/P.t.t*, orange AB046525 *P.t.t* and purple AF498266 *P.t.s* **2(b).** Tree Order Scan of HBV sequences, indicating positions of individual sequences (y axis) in phylogenetic trees generated from sequential 250-base sequence fragments, incrementing by 50 bases. Changes in sequence order as a result of changes in phylogeny at the 70% bootstrap level are shown. Sequences are colour coded by host species and sub-species of chimpanzee, as indicated by the labels in left and right margin: *Gorilla gorilla*, blue (Gor); *Pan troglodytes troglodytes*, yellow (*Ptt*); *Pan troglodytes ellioti*, green (*Pte*); *Pan troglodytes verus*, purple (*Ptv*); *Pan troglodytes schweinfurthii*, violet (*Pts*); and *Hylobates pileatus* (*Hyl*) (out-group-line 1-GII), red. For comparison the Tree Order Scan has been aligned with scale genome of HBV (top panel). Recombinant sequences are highlighted as by dashed lines; black gorilla/*P.t.e* ECO50003LIP3, green FJ798099 *P.t.e/P.t.t*, brown FJ798098 *P.t.e/P.t.t*, orange AB046525 *P.t.t* and blue AF498266 *P.t.s.*

Phylogenetic trees of 200 bp fragments incrementing by 50 bp across the entire genome were constructed to identify changes in phylogeny potentially indicative of recombination events. This procedure was automated by the program TreeOrder Scan [22] in SSE v1.0 [Manuscript in preparation]. The tree position of each sequence across the genome is recorded (y-axis) and colour-coded by HBV genotype and species (Fig. 2a) and by sub-species (Fig. 2b). Changes in the tree order of individual sequences or genotypes with 70% or greater bootstrap support are indicative of alterations in the phylogenetic relationships of clades and identify potential recombination breakpoints.

Consistent with previous findings [22], phylogenetic relationships between human genotypes changed between genome regions, leading to alterations in the branching order. For example, genotypes D and E were largely phylogenetically distinct across the genome, but between position 1950 and 2500 (the core gene); genotype E falls within the genotype D clade. Excluding gorilla and chimpanzee sequences, recombination events typically occur around positions 750, 900, 1600, 1950, 2500, 2650 and 2750, frequently coinciding with gene boundaries as previously described [22].

In order to detect recombination events between HBV variants from different ape taxa, a Tree Order Scan was performed with all HBV reference and study sequences from gorillas and chimpanzees with a *Hylobates pileatus* sequences as an out-group (Fig. 2b). Outside of the core gene region, gorilla-derived variants were phylogenetically distinct from other ape-associated and human genotypes. However, between positions 1560 and 2120 (in the X and pre/core gene), the gorilla isolate ECO50003LIP3 sequence grouped within the chimpanzee clade, indicative of a recombination event. ECO50003LIP3 showed 99.45% (99.0–99.9%) similarity with other gorilla variants between positions 1 and 1559 and 99.65% (99.5–99.8%) similarity between positions 2121–3272, and an average 95.5% and 97.4% similarity with *P.t.ellioti* variants across the same respective regions.

This analysis also identified recombination in the sequence A498266 (Fig 2b), a *P. t. schweinfurhii* HBV isolate previously identified as a recombinant between human genotype C and chHBV [18]. For this sequence, a 500 nt region between positions 550–1050 nt grouped with species C while the remainder of the genome grouped with *P.t.troglodytes* sequences, consistent with previous findings [18]. The recently described HBV sequence from *P.t.schweinfurthii* [19] may therefore represent the “original” *P. t. schweinfurthii* sequence, from which the recombinant arose [19]. The inclusion of these sequenced isolates described in our investigation supports the formation of a *P. t. schweinfurthii* species-specific clade (Fig. 1) which includes the genotype C/chHBV recombinant. Corroborating evidence of recombination was identified in Cameroon chimpanzee sequences, FJ09898.1 and FJ09899.1; between positions 820 and 1300 nt, traversing a partial region of the polymerase gene confirming previous findings on recombinant *P.t.t/P.t.e* HBV variants [21].

**Figure 3:**
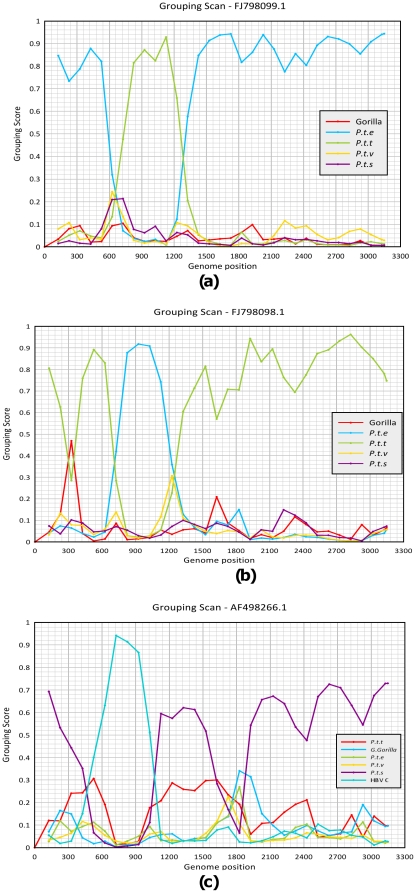
Grouping Scan analysis. Sequence fragments of 250 bases incrementing by 100 bases with 100 bootstrap replicates, were used to compare and analyse (a) *P.t.troglodytes/P.t.ellioti* recombinant FJ98098.1 (b) *P.t.ellioti/P.t.troglodytes* recombinant FJ98099.1 (c) *P.t.schweinfurthii* isolate A498266; (d) *P.t.troglodytes* AM117396 (e) *P.t.troglodytes* recombinant AB046525 (f) study recombinant *Gorilla gorilla* HBV sequence (ECO50003); to sequence groups from *Gorilla gorilla* (red), *Pan troglodytes ellioti* (blue), *Pan troglodytes troglodytes* (green), *Pan troglodytes verus* (yellow), *Pan troglodytes schweinfurthii* (purple) and human genotype HBV/C (light blue) with respect to A498266. Values >0.5 indicate clustering within the indicated group.

To confirm the position and phylogenetic grouping of the putative recombinant sequences identified by the TreeOrder scan, a Grouping Scan was performed [22]. This examines how deeply embedded the test sequence is within clades formed by non-recombinant control sequences assigned into species-associated groups (*P.t.e*, *P.t.t.*, *P.t.v* and *Gorilla gorilla*) (Fig. 3). This method identified two changes in grouping of the query sequence, ECO50003LIP3 at position 1560 where it changed grouping from the gorilla HBV clade to the *P. t. ellioti* sub-species clade and a reversion to the gorilla clade at position 2120. Grouping scan analysis of recombinant sequences A498266 and FJ98098.1 provides substantial support for the formation of recombinant regions between positions 550–1050 nt and 820–1300 nt respectively. However, the Tree Order or grouping scan methods provided no evidence for recombination in the *P.t.troglodytes* derived sequence, AM117396 (based on its grouping in Fig. 1a) between chimpanzee sub-species.

Sequence AB046525 from *P.t.troglodytes* in Central Africa grouped separately from other *P.t.t* variants in core gene region, consistent with past recombination with a divergent and currently uncharacterised genotype of HBV [23]. Both TreeOrder scan analysis and Grouping Scan analysis confirmed the rest of the genome groups consistently with *P.t.troglodytes*, while adopting an outlier position to all other chimpanzee and gorilla isolates in the core region.

## Discussion

In this study a large number plasma samples from great apes and monkeys from Cameroon were screened for HBV-DNA. The prevalence amongst chimpanzees in our study was found to be 9.7% (6/62), 18% (2/11) in gorillas. This confirms previous findings on the existence of HBV in great apes in the wild [16], [22] and the rates are similar to those observed in human populations in areas of endemic infection, such as Central Africa and South East Asia. In a recent study, the prevalence of active HBV infection (DNA-positive in plasma) was 15% (8/53) in gorillas and 18% (40/205) in chimpanzees [15], [24].

Eight new complete HBV genomes were obtained in the current study from two gorillas and six chimpanzees born in the wild. Gorilla sequence ECO50065LIP3 was almost identical to the previously described sequence, FJ798095 [21], and retrospective analysis revealed that these sequences originated from the same animal in Limbe Wildlife Centre. The six complete chimpanzee HBV sequences all grouped with previously identified *P.t.ellioti* variants [21] although two were recovered from *P.t.troglodytes*. Current analyses cannot determine whether these two cross species infections occurred in the wild or through contact with infected *P.t.ellioti* chimpanzees while in captivity although the latter is highly likely given the mixing of chimpanzee subspecies in sanctuaries. The existence of *P.t.troglodytes* and *P.t.ellioti* associated variants of HBV, as is the case for other chimpanzee subspecies (*P.t.verus* and *P.t.schweinfurthii*) requires further investigation of variants infecting chimpanzees in the wild in Cameroon, in particular in regions where these sub-species may converge, for example around the confluence of the Mbam and Sanaga Rivers [25]. The observation of cross-species infections and recombination events for HBV infections also provides an additional reason for ensuring that captive chimpanzees are correctly identified to subspecies and segregated appropriately to avoid the creation of recombinant HBV variants with potentially different pathogenicities and transmission patterns.

The phylogenetic tree comparing these eight sequences to previously recorded HBV sequences in non-human primates confirms that variants recovered from chimpanzees and gorillas in Africa are distinct from those reported in Asian gibbons and from all human HBV sequences that cluster separately into genotypes A–H. This is consistent with the geographical association of HBV in NHP previously reported [24]. Phylogenetic analysis based on complete genome found significant bootstrap support for the formation of four HBV clusters that, excluding likely cross-species transmissions, corresponded with *P. troglodytes* subspecies: *P. t. troglodytes* (81% bootstrap), *P. t. verus* (100% bootstrap), *P. t. ellioti* (100% bootstrap) and *P. t. schweinfurthii* (100% bootstrap). Recent phylogenetic analysis of the *P. t. schweinfurthii* HBV strain; confirmed by our GroupScan analysis; (Fig. 3c) showed evidence of interspecies recombination between HBV infecting chimpanzees and the human HBV-C genotype strain [6], [18]. Phylogenetic trees of the recombinant region and equivalent fragments either side, inclusive of all reference and study sequences, confirm the sub-species association of HBV in NHPs (Fig. 1b to 1d). The phylograms also support the recombinant data of the Tree order and Grouping scan analysis, with respect to the location and confidence level for the recombinant region and sequence (Fig. 1b). The correlation of HBV sequences with the different subspecies of chimpanzees indicates either that the HBV strains and their hosts have co-evolved or alternatively have diverged through allopatric separation.

The co-divergence hypothesis for the distribution of non-human HBV genotypes in Africa and South East Asia presupposes that the distinct variants of HBV found in different ape species and subspecies arose during the period of their evolution, over the past 5-7 million years. However, such a hypothesis implies an extraordinarily slow maintained substitution rate of HBV; the 5% divergence between gorilla and chimpanzee variants requires a minimum substitution rate of 5×10^−9^ substitutions per site per year (SSY), slower even than mammalian coding region substitution rates and quite distinct from the 10^−4^–10^−5^ SSY rates estimated for HBV over shorter periods [26]. The co-divergence hypothesis would additionally predict that variants infecting gorillas should be more divergent from and take an outlier phylogenetic position to the subspecies-associated variants of chimpanzees that would have diverged from each other between 0.8–1.5 million years ago [24]. This is clearly not the case in the phylogenetic analysis shown in Fig. 1, where the gorilla clade adopts an internal branching position among chimpanzee sub-species associated variants. Similarly, the co-divergence hypothesis cannot explain the inlier position of orangutan derived HBV variants within the gibbon clade [27], nor the substantial sequence diversity in HBV variants infecting humans and their outlier position to NHP-derived HBV sequences. On the other hand, co-divergence of virus and host and the implied extremely low long-term viral substitution rates have been observed within other primate associated viruses, including simian immunodeficiency virus (SIV) and simian foamy virus [15], [24]. In the case of HBV, substitutions may accumulate slowly as a result of the extreme constraints on sequence change in the HBV genome imposed by the extensive use of overlapping reading fames for protein coding, as well as RNA secondary structures required for genome transcription and translation [22], [24]. However, co-divergence does not explain how HBV-like viruses infecting rodents, squirrels and birds could have become so divergent from human and primate variants over period perhaps only 10–20 times as long as the period of ape divergence.

The alternative hypothesis which could account for the pattern of sequence diversity of HBV in NHPs is divergence through allopatric separation but ongoing transmission of HBV between ape species and subspecies. This alternative hypothesis would also account for the internal branching position of the gorilla clade within the chimpanzee derived HBV sequences. As previously discussed, it also accounts for the inlier position of orangutan-derived sequences deep within the gibbon clade, and their close genetic relationship with the sympatric *Hylobates agilis* gibbon species. These two species occupy proximal or overlapping habitats in Borneo, while HBV variants infecting gibbons from elsewhere in Asia group separately [24], [28]. The lack of sequence diversity between infected orangutans implies a more recent introduction of HBV into this species.

Detection of recombinants efforts might therefore focus on geographical regions where different non-human primate species and sub-species come into contact, for example the upper reaches of the Sanaga River in Cameroon *P.t.ellioti* and *P.t.troglodytes* may occasionally mix [25] and southern Cameroon where *P.t.troglodytes* and *G.gorilla* distributions overlap. The later hypothesis may potentially explain why no recombinant variants have been detected in *P.t.verus*, a subspecies found in West Africa that is geographically isolated from other non-human ape species. The timescale for the proposed geographical isolation of HBV variants infecting different NHP species and sub-species is not known. If we take the substitution rate for HBV measured over short periods [29], the introduction and geographical differentiation of HBV in African apes may have occurred relatively recently indeed, approximately 5,000 years using the previously described substitution rate of 10^−5^ substitutions per site per year [26], [30].

Recombination affecting a short region between either end of the polymerase gene (partial X gene and Pre-core/core) in one *Gorilla gorilla* isolate is the first recorded occurrence of recombination between chimpanzee- and gorilla-derived HBV variants. The recombination event between the gorilla and *P.t.ellioti* variants likely occurred in the wild as gorillas and chimpanzees are never co-housed in captivity. The position of the breakpoint region is close to several documented previously in human genotypes [22] (Fig. 2a). A recent study of duck HBV [31] recorded a similar breakpoint event between position 1010–2304 bp, incorporating the region of the X gene, which is believed to promote cell growth and inactivate growth regulating molecules [32], [33].

Complex epidemiological factors such as transmission routes affect the pool of circulating HBV variants; however their spread may be enhanced by the evolution of recombinant variants, allowing the virus to transmit more efficiently between species. Phylogenetic studies have previously indicated that recombination events in HBV are quite common [12], [22] and recombinant strains have been shown to possess distinct biological features and produce different clinical outcomes compared to their parental strains [31]. Further work is required to investigate the distribution of HBV recombinants in Cameroon, their potential impact on host species and the evolution of HBV in NHPs in the wild.

The evidence of animal reservoirs, cross-species transmission and recombination between human and ape HBV variants have important implications for the eradication of HBV worldwide. The transmission of recombinant gorilla/chimpanzee HBV and endemic infection among apes in the wild will hinder efforts to eradicate HBV globally, particularly in regions where apes and humans come into contact [2]. Further sampling of isolated populations of NHPs and in areas of sympatry is required to further investigate the currently conflicting evolutionary hypotheses for HBV diversity and to determine how and when HBV spread between Africa and Asia. Understanding the relationship between human and NHP HBV variants will aid in resolving this question and explain the possible role humans have played in disseminating HBV globally.

## Materials and Methods

### Samples

A total of 164 non-human primate plasma samples were screened for the presence of HBV DNA. Samples were collected from animals at three wildlife sanctuaries in Cameroon between August 2004 and August 2009. Animals were brought to the sanctuaries following confiscation by the authorities or abandonment by owners. All chimpanzees and gorillas sampled were wild-born, while other species included both wild and captive-born individuals. *Gorilla gorilla* and *Pan troglodytes* were generally housed separately in the sanctuaries, while some species of monkeys were housed in mixed groups. However, the captive history of some animals is unknown as some were held in captivity prior to their arrival in the sanctuaries and the sanctuaries themselves were not always under the current management.

Blood samples were collected via venepuncture from 73 apes comprising 11 gorillas (*Gorilla gorilla*) and 62 chimpanzees (*Pan troglodytes troglodytes* and *Pan troglodytes ellioti*), and from a variety of Old World Monkey species: *Cercocebus agilis* (*n* = 7), *C. torquatus* (*n* = 2), *Cercopithecus cephus* (*n* = 3), *C.erythrotis* (*n = *4), *C. l'hoesti preussi* (*n* = 4), *C. mona* (*n* = 9), *C. nictitans* (*n* = 3), *C. pogonias* (*n* = 1), *C. tantalus* (*n* = 3), *Erythrocebus patas* (*n* = 3), *Lophocebus albigena* (*n* = 5), *Mandrillus leucophaeus* (*n* = 20), *M. sphinx* (*n* = 9), and *Papio anubis* (*n = *20). Plasma was separated by centrifugation and frozen at −80°C until testing. Samples were shipped to the United Kingdom from Cameroon in compliance with UK and Cameroon laws and the Convention on International Trade in Endangered Species of Wild Fauna and Flora (CITES) [34].

### HBV Screening

DNA extractions were performed with 50 µl of plasma using the AllPrep DNA/RNA minikit (Qiagen) according to the manufacturer's instructions with DNA eluted in a final volume of 50 µl. Samples were screened by nested PCR using first round primers S1, S5 and second round primers S3 and S6 as previously published [2] .Three µl of extracted DNA was then amplified in a PCR mixture containing Promega Access reagents (Promega, Chilworth, Southampton, United Kingdom). First-round amplification involved 30 cycles of 94°C for 18 s, 55°C for 21 s, and 72°C for 1.5 min; and 1 cycle of 72°C for 5 min. One µl of first round PCR product was amplified further in a second-round PCR using the internal primers and conditions previously described [2]. PCR positive samples with screening primers included samples from 9 apes (n = 2 gorillas, n = 7 chimpanzees) and 2 Old World Monkeys. The entire HBV genome was successfully sequenced for 2 gorillas (ECO50003LIP3 and ECO50065) and 6 chimpanzees (ECO50083, ECO50210, ECO51109, ECO51212, ECO51394 AND ECO51377) in overlapping fragments using primers as published [2] in addition to new primer sets: Set 1: Outer Sense 49 (CTGGATGTGTCTGCGGCGTT position 375) and Outer Anti-Sense 51 (GCACAGACGGGGAGACCGCG position 1542) followed by Inner Sense 48 (CCAATTTGTCCTGGYTATCG position 395) and Inner Anti-Sense 50 (TAAAGAGAGGTGCGKCCCGT position 1522), Set 2: Outer Sense 52 (CWTTRTATGCATGTATACAAGC position 1082) and Outer Anti-Sense 55 (GGCTTCMCGGTACARAGCTGA position 2054) followed by Inner Sense 53 (TCGCCAAYTTAYAAGGCCTT position 1121), and Inner Anti Sense 54 (GCGGTGTCRAGRAGARCACG position 2033), and finally Set 3: Outer Sense 56 (TTGCCTKCTGAYTTCTTTCC position 2025) and Outer Anti-Sense 59 (CCCATGCTGTAGCTCTTGTTCC position 2888), followed by Inner Sense 57 (CGTGATCTYCTYGACACCGC position 2052) and Inner Anti Sense 58 (CAAGAATATGGTGACCCACA position 2868). First round PCR involved 35 cycles of 94°C for 18 s, 55°C for 21 s, and 72°C for 1.5 min; and 1 cycle of 72°C for 5 min, followed by second round PCR of 40 cycles at matching conditions. Touch-down PCR between 65°C and 50°C with 0.5°C decline per cycle was also applied.

Mitochondrial sequencing to confirm host species and sub-species for the 9 complete genomes was carried out using primate specific primers: Forward: PrCOI: CTATTYGGYGCATGAGCNGG Reverse: PrCOI: TARAAGAARGTRGTRTTRAGGTTRC, followed by Forward: PrCOI CAGCCCTAAGYCTYCTYATTCG and Reverse: PrCOI GAYDGATCAGACRAAYARGGG (Where R: A/G, Y: T/C, D: G/A/T and N: G/A/T/C). First and second round PCR conditions involved 30 cycles of 94°C for 22 s, 50°C for 24 s, 72°C for 1.5 min; and 1 cycle of 72°C for 5 min.

### Sequencing of PCR products and sequence analysis

Positive second round PCR amplicons were sequenced in both directions using the inner sense and inner antisense primers used in the second round of amplification. Sequencing was executed using Big Dye Terminator v3.1 (Applied Biosystems) according to the manufacturer's instructions. Sequences were read at the Gene Pool facility (University of Edinburgh) and analyzed using SSE v1.0 software. Sequences obtained in this study have been assigned the GenBank accession numbers JQ664502–JQ664509.

Phylogenetic trees were constructed using a bootstrap neighbour-joining method with 100 replications and incorporating the Kimura-2-Parameter model of nucleotide substitution and a uniform rate variation among sites using the MEGA 5.01 [35] software package with pairwise deletion for missing data. Tree construction involved two datasets of 31 complete HBV genome sequences from *Pan t. troglodytes*, *Pan t. ellioti*, *Pan t. verus*, *Pan t. schweinfurthii* and *Gorilla gorilla* GenBank sequences and final phylogenetic analysis that included representative human HBV sequences of genotypes A–H.

Recombination analysis was carried out using Tree Order Scan package of SSE v1.0 [Manuscript in preparation] generating an image of individual sequence positions in phylogenetic trees generated from sequential 250-base sequence fragments, incrementing by 25 bases. Changes to the sequence order due to changes in phylogeny at the 70% bootstrap level are reported.
